# Collaborating across government agencies to combine linkage processes and algorithms to create an Australian National Linkage Spine (NLS) to facilitate cross-jurisdictional linkage

**DOI:** 10.23889/ijpds.v9i5.2818

**Published:** 2024-09-18

**Authors:** Theresa Nunan, Elena Ougrinovski, Brett Frazer, Kelly Dahl, Bronwyn Wilson, Rian Jenkins, Clinton Paine

**Affiliations:** 1 Australian Bureau of Statistics; 2 Australian Institute of Health and Welfare

The Australian Bureau of Statistics (ABS) and the Australian Institute of Health and Welfare (AIHW) are co-technical lead agencies for developing the National Disability Data Asset (NDDA) which will bring together data across jurisdictions to build a more complete picture of the lives of people with disability. ABS and AIHW are collaboratively building a National Linkage Spine (NLS), a high-quality high-coverage population dataset to be shared under new legislation to facilitate cross-jurisdictional linkage and build the NDDA.

ABS and AIHW each have extensive data integration experience, however our linkage methods have evolved differently in response to the needs of our respective researcher client bases and the datasets we work with. An ABS-AIHW cross-agency team was established to align terminology and share data processing methods, linkage approaches, and locally built linkage tools. The team collaborated on the design of an NLS that combines the best aspects of each of our approaches.

The NLS build methodology delivers aligned processing of input data and applies a combination of ABS’s deterministic linkage algorithm and AIHW’s probabilistic linkage and machine learning algorithms. The NLS was peer reviewed by its future jurisdictional users and is considered fit for purpose for delivering the NDDA.

The open sharing of knowledge and tools has significantly benefited both agencies and the broader NDDA project. Takeaways from this project extend beyond it, with improved data process approaches being considered by each agency in other work, and a growing common agreement of linkage best practice.

